# Ovarian Steroid Cell Tumour: Correlation of Histopathology with Clinicopathologic Features

**DOI:** 10.4061/2011/987895

**Published:** 2011-03-02

**Authors:** Ghazala Mehdi, Hena A. Ansari, Rana K. Sherwani, Khaliqur Rahman, Nishat Akhtar

**Affiliations:** ^1^Department of Pathology, Jawaharlal Nehru Medical College, Aligarh Muslim University, Aligarh 202002, India; ^2^Department of Obstetrics and Gynaecology, Jawaharlal Nehru Medical College, Aligarh Muslim University, Aligarh 202002, India

## Abstract

Ovarian steroid cell tumours (not otherwise specified) are rare neoplasms of the ovary and are classified under lipid cell tumours. Their diagnosis can be considered as one of exclusion. Histopathologically, the tumour should carefully be evaluated for microscopic features of malignancy, but it is essential for the clinician and the pathologist to remember that in these tumours, pathologically benign histomorphology does not exclude the possibility of clinically malignant behaviour. Our case study focuses on the comparative findings in a postmenopausal female diagnosed with an ovarian steroid tumour (not otherwise specified). A careful correlation between clinical and surgical evaluation and microscopic analysis is necessary, as is a regular followup.

## 1. Introduction

The spectrum of ovarian neoplasms covers an extremely wide range of tumours. The best recognized of these are the surface epithelial cell tumours. Amongst the less common variants, lipid or steroid cell tumours comprise an important category, although they account for only 0.1% of all ovarian tumours [1] 

The origin of steroid cell tumours has long been a matter of controversy and debate. Their nomenclature as such arises from their resemblance to steroid hormone secreting cells (lutein/leydig/adrenal cortical rest cells) [2] and they have been basically subclassified as stromal luteomas, leydig cell tumours, and steroid cell tumours, not otherwise specified [3]. The term steroid cell tumours (not otherwise specified) was first described by Scully [4, 5] and signifies that the cell lineage is not defined, and they cannot be categorized as either stromal luteomas or leydig cell tumours [3]. However, the majority of cases fall under this category [3, 4].

The following report focuses on a case of a steroid cell tumour (not otherwise specified) diagnosed in a 54-year-old postmenopausal female. The case was significant keeping in mind the short duration of complaints, the age of the patient, lack of any overt androgenic manifestations, and discrepancies between clinicopathological findings and light microscopic features.

## 2. Case Report

A 54-year-old multiparous postmenopausal female presented in the outpatient section of the department of obstetrics and gynecology in our hospital, with a short history of pain in the abdomen (in the epigastric region) for two months, associated with abdominal distention and loss of appetite for fifteen days. There were no other associated complaints. 

Perabdominal examination revealed distention of the abdomen. Pervaginal examination did not yield definite findings, with a vague fullness being detected in both fornices. Uterine size could not be made out. 

**Figure 1:**
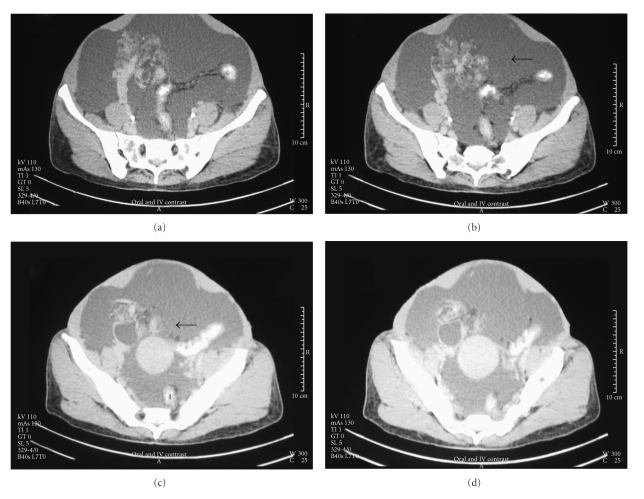
CT Scan—Heterogenous, solid-cystic, moderately enhancing right adnexal mass (↑).

A CT scan was carried out which showed a heterogenous, solid-cystic, right adnexal mass (Figure 1), associated with normal uterine size and a leiomyoma in the posterior wall of the uterus. Free fluid was present in the peritoneal cavity.

The patient underwent an exploratory laparotomy, with total abdominal hysterectomy, bilateral salpingo-oopherectomy and sampling of pelvic and mesenteric lymph nodes and omentum. Imprint/scrape smears were prepared peroperatively from the tumour surface, hemidiaphragm, liver, and peritoneum and from what appeared to be deposits on the omental and colonic surface. Fluid samples were also collected from the subdiaphragmatic spaces and para-colic gutters and pouch of Douglas. We did not attempt to assess the tumour intraoperatively with frozen sections. 

The resected specimens were sent to the histopathology section of the department of pathology, while the smears and fluid samples were received in the cytopathology section and were processed as per the protocol.

### 2.1. Gross Examination

**Figure 2:**
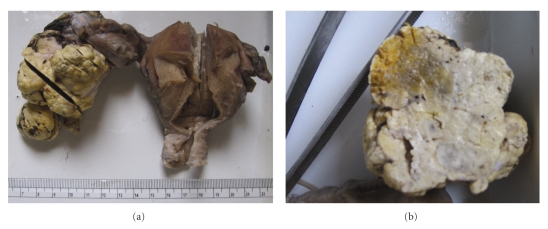
Gross Appearance. (a) Specimen of resected uterus, cervix and bilateral tubes and ovaries, with a large nodular solid tumour in right ovary. (b) Cut surface of tumour showing a predominantly solid, yellowish appearance with occasional small cystic spaces.

 A firm yellow-coloured tumour was seen at one end of the right ovary, 6 × 5 × 4 cms in size, with a nodular/bosselated surface. The cut surface was solid with occasional small cystic spaces, yellowish and with focal areas of haemorrhage. There was capsular extension with presence of satellite nodules. The gross appearance is demonstrated in Figure 2.

 The rest of the ovary was occupied by a smooth cystic cavity, 4 × 3 cms, containing watery fluid. The right fallopian tube was grossly normal, as were the left tube and ovary. An intramural leiomyoma, 1.5 cms in diameter, was detected in the posterior and lower segment of the uterus. Lymphnode and omental samples were also processed.

### 2.2. Light Microscopy

**Figure 3:**
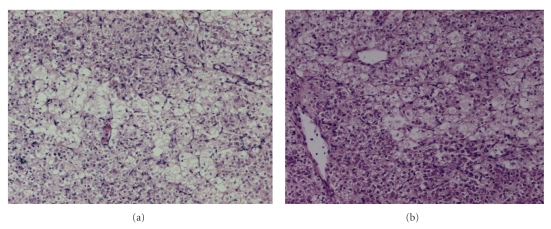
(a) and (b) Bimodal tumour cell population; large polyhedral cells with vacuolated cytoplasm and smaller cells with eosinophilic/granular cytoplasm in a vascular stroma (H&E ×125 ).

**Figure 4:**
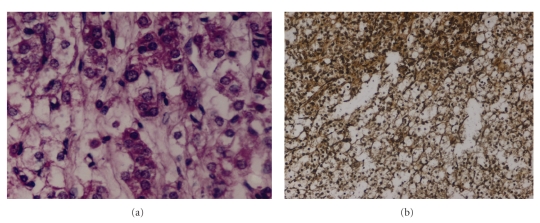
(a) Tumour cells showing vesicular nuclei with nucleoli and nuclear atypia; mitotic figures are not seen (H&E ×500). (b) Diffuse presence of fine reticulin fibres around small cell groups (Reticulin stain ×125).

On evaluation of the light microscopic features, the tumour areas showed a bimodal cell population; there were large, round to polyhedral cells with vacuolated cytoplasm as well as smaller cells which had eosinophilic granular cytoplasm (Figure 3). Some degree of overcrowding, overlapping and nuclear atypia was clearly evident in these smaller cells (Figure 4(a)). The nuclei were predominantly vesicular and nucleoli were present in the small cells. Reinke's crystals were not observed. Mitotic activity or necrosis was not seen in the sections examined from the tumour site. The stroma was loose with several vascular channels randomly distributed between the tumour cells. A reticulin stain was applied and the resultant slide showed fine reticulin-positive fibres around small groups of 2-3 tumour cells throughout the tissue (Figure 4(b)). The presence of glycogen was excluded with the help of a negative Periodic acid-Schiff reaction.

The tumour tissue was also sent for immunohistochemical analysis for calretinin but the result was negative.

The associated cystic area in rest of the ovary turned out to be a simple serous cyst. Other histopathological findings included chronic cervicitis, an atrophic endometrium with endometritis, myometritis, and an intramural leiomyoma, salpingitis, and a simple serous cyst in the left ovary.

 Reactive changes were noted in the mesenteric lymph node, while the sample of pelvic lymphnode yielded only fibrofatty tissue. No tumour cell deposits were detected on light microscopy in the sections of lymphnode and omentum.

The cytologic samples (fluid and imprint smears) did not yield any significant findings.

The histopathological features supported a diagnosis of a Lipid (Steroid) cell tumour (not otherwise specified) of the right ovary with serosal nodules. 

The patient was treated postoperatively with chemotherapy and is on regular followup.

## 3. Discussion

The most important factor to be determined in lipid/steroid cell tumours of the ovary is whether the tumour has malignant features or not. In one of the major studies done on lipid cell tumours of the ovary [4], certain histopathological findings were found to correlate highly with clinically malignant behaviour. These can be summarized as 2 or more mitotic figures per 10 high power fields (92%), necrosis (86%), a diameter ≥7 cms (78%), haemorrhage (77%), and grade 2-3 atypia (64%). 

It is interesting to note that in the case under discussion, the patient had ascites and on gross examination, there was evidence of capsular extension and satellite nodules.

In contrast to the clinical findings, the microscopic appearance did not reveal any prominent finding in favour of malignancy. Mitosis was not noted, there was no haemorrhage or necrosis, tumour size was less than 7 cms in all dimensions, and only grade 1 atypia was observed. This tumour thus had a benign microscopic appearance. 

It is known that pathologically benign steroid cell tumours can behave in a clinically malignant fashion [6]. Therefore, careful followup is essential in such cases which do not have clinical or pathological evidence of malignancy. As such, presence of metastasis may be the only definite evidence of malignant behavior [1]. Our patient has also been placed on regular followup, after treatment with surgery and chemotherapy.

 Another unusual finding in this case is the apparent absence of androgenic manifestations. However, it is possible that the changes were very subtle and not clinically evident. 

Some of the other pathological entities that have to be excluded are leydig cell tumors, luteinized granulosa cell tumours, clear cell carcinomas, and metastatic renal cell carcinomas. Reinke's crystals can usually be demonstrated in leydig cell tumours. Granulosa cell tumours can have prominent luteinized areas but typical foci of the tumour can be identified. A Periodic acid-Schiff stain can be used to demonstrate the presence of glycogen in clear cell carcinomas and metastatic renal cell carcinomas, which is absent in steroid cell tumours.

A calretinin stain was performed in this case but was found to be negative. These tumours are usually positive for inhibin although it is not specific for this tumour. A recent study analysed ovarian sex-cord stromal tumours for calretinin positivity [7]. All the six steroid cell tumours in this study were positive for the marker. Calretinin can be considered a sensitive marker for ovarian sex-cord stromal tumours in general, but is less specific [8]. A similar study has evaluated the applicability of CD56 as a marker in ovarian sex-cord stromal tumours [9]. The authors concluded that it is a sensitive marker for these neoplasms, although it cannot help to differentiate a sex-cord stromal tumour from a neuroendocrine neoplasm. A number of immunohistochemical markers were reviewed for their utility in differential diagnosis of sex-cord stromal tumours and inhibin and calretinin were found to be most useful, among others, in differentiating sex-cord stromal from nonsex-cord stromal tumours, while MART-1 was diagnostically useful for steroid cell tumours [10].

A careful correlation between clinical findings and histopathology is always essential, as is amply demonstrated by this particular case. Moreover, the necessity of a regular followup must be stressed upon, with the aim of detecting a possible recurrence or evidence of distant metastasis.
